# Mating system is correlated with immunogenetic diversity in sympatric species of Peromyscine mice

**DOI:** 10.1371/journal.pone.0236084

**Published:** 2020-07-23

**Authors:** Jesyka Meléndez-Rosa, Ke Bi, Eileen A. Lacey

**Affiliations:** 1 Department of Integrative Biology, University of California, Berkeley, California, United States of America; 2 Museum of Vertebrate Zoology, University of California, Berkeley, California, United States of America; 3 Computational Genomics Resource Laboratory, University of California, Berkeley, California, United States of America; University of Iceland, ICELAND

## Abstract

The number of reproductive partners per individual varies markedly across animal mating systems. This variation may be an important determinant of patterns of immunogenetic diversity, particularly at Major Histocompatibility Complex (MHC) Class I and II loci. To compare immunogenetic variation in taxa with markedly different mating systems, we used RNAseq-generated data to quantify genotypic diversity in three species of Peromyscine rodents: the monogamous California mouse (*Peromyscus californicus*) and the polygynandrous deer mouse (*P*. *maniculatus)* and brush mouse *(P*. *boylii*). By sampling populations of these species from multiple localities in California, we were able to conduct replicated analyses of the relationship between mating system and immunogenetic variation. Across the four localities sampled, diversity at MHC Class I and II genes was consistently higher in the two polygynandrous species. We found no evidence that sampling location (i.e., variation in habitat conditions) contributed to observed differences in MHC variation among populations or species. Collectively, our data indicate that immunogenetic variation in Peromyscine mice is associated with reproductive behavior, rather than geographic locality or habitat type. The consistently greater variability detected in the polygynandrous species examined suggests that balancing selection imposed by behaviorally-mediated pathogen exposure is important in maintaining variation at MHC genes in these animals.

## Introduction

Mating systems are complex behavioral phenotypes, the genomic implications of which are generally poorly understood [1,2]. Among vertebrates, studies that explore interactions between reproductive behavior and patterns of genetic variability have often focused on the genes of the Major Histocompatibility Complex (MHC), an emphasis that reflects the critical role of these genes in the adaptive immune response [3,4]. The typically high levels of variability at these loci are thought to arise due to strong balancing selection [3,4] driven by exposure to pathogens [5,6], a relationship that may be amplified by mate choice decisions that enhance MHC diversity among offspring [7]. Consistent with this, mating systems that expose individuals to a greater number or diversity of pathogens are expected to result in stronger balancing selection and increased diversity at MHC loci [8,9]. These relationships should be particularly evident when comparing monogamous systems in which each individual mates with only a single partner to polygynandrous systems in which individuals of both sexes mate with multiple partners [10,11]. Specifically, selection on and variability at MHC genes should be greater in polygynandrous animals due to the greater potential for pathogen transmission associated with interactions with multiple reproductive partners.

Despite clear expectations regarding the effects of mating systems on immunogenetic variability, few studies have examined these relationships in natural populations of vertebrates (but see [9,12]). Those studies that have considered mating systems have typically focused on one to a few MHC loci, with the result that they may not provide a comprehensive picture of how patterns of reproductive behavior and pathogen exposure interact to affect genetic variability. Further, these studies have typically focused on single populations of animals, despite compelling evidence that local habitat conditions affect both mating system [10,13] and pathogen exposure [4]. As a result, the generality of relationships between mating systems and MHC variation have not been assessed across populations of conspecifics, particularly those living in different habitats.

To explore how differences in mating system shape variation at multiple MHC loci, we compared levels and patterns of immunogenetic diversity in three species of *Peromyscus* mice that occur in California. The California mouse (*P*. *californicus*) occupies oak savannah and chaparral habitats from the San Francisco Bay to northern Baja California [14,15]. Behaviorally, this species is unusual in that it is both socially and genetically monogamous [16,17], meaning that each adult engages in reproductive interactions and produces offspring with only a single member of the opposite sex. In contrast, the brush mouse (*P*. *boylii*) and the deer mouse (*P*. *maniculatus*) are both polygynandrous, with individual males and females mating with multiple partners [18,19]. Because these three species co-occur throughout much of coastal California, they provide an important opportunity to characterize genetic diversity in relation to mating system across distinct populations subject to different environmental conditions.

To capitalize on this comparative system, we used transcriptomic sequencing to quantify variation at multiple MHC genes in these species. Specifically, we sought to test the prediction that monogamy in *P*. *californicus* is associated with reduced variability and less intense selection at MHC loci compared to sympatric populations of the polygynandrous *P*. *boylii* or *P*. *maniculatus*. Because the samples analyzed had been obtained from four geographically and ecologically distinct locations in coastal and inland California, we also sought to determine if differences among the study species varied with sampling locality, as expected if local differences in pathogen communities influence selection patterns on MHC loci [20]. Collectively, these analyses provide a particularly comprehensive analysis of patterns of variability at MHC genes and generate important new insights into the behavioral factors contributing to immunogenetic variation in natural populations of mammals.

## Materials and methods

### Study species

We examined patterns of immunogenetic diversity in three species of *Peromyscus* that occur in California. *P*. *maniculatus* is a small-bodied (10–24 g), ecologically generalized species that occurs in a wide range of habitats in California, including deciduous woodlands, deserts, coastal scrub, chaparral, and grasslands [21,22]. *P*. *boylii* is larger-bodied (22–36 g) and more ecologically restricted, occurring only in chaparral and scrub forest [21,22]. *P*. *californicus*, the largest of the study species (32–54 g), is also found in chaparral and scrub forest habitats from the San Francisco area to northern Baja California [21,22]. *P*. *maniculatus* and *P*. *boylii* are polygynandrous, meaning that each individual mates with multiple partners of the opposite sex [18,19]. In contrast, *P*. *californicus*, is both socially and genetically monogamous [17], indicating that each individual mates with only a single animals of the opposite sex. Phylogenetically, *P*. *californicus* and *P*. *maniculatus* are more closely related than *P*. *maniculatus* and *P*. *boylii* [23] and thus inclusion of the latter species allows for a more robust assessment of the role of mating system in generating patterns of immunogenetic variation in free-living mammals.

### Field sampling procedures

#### Field sites

**Fig 1:**
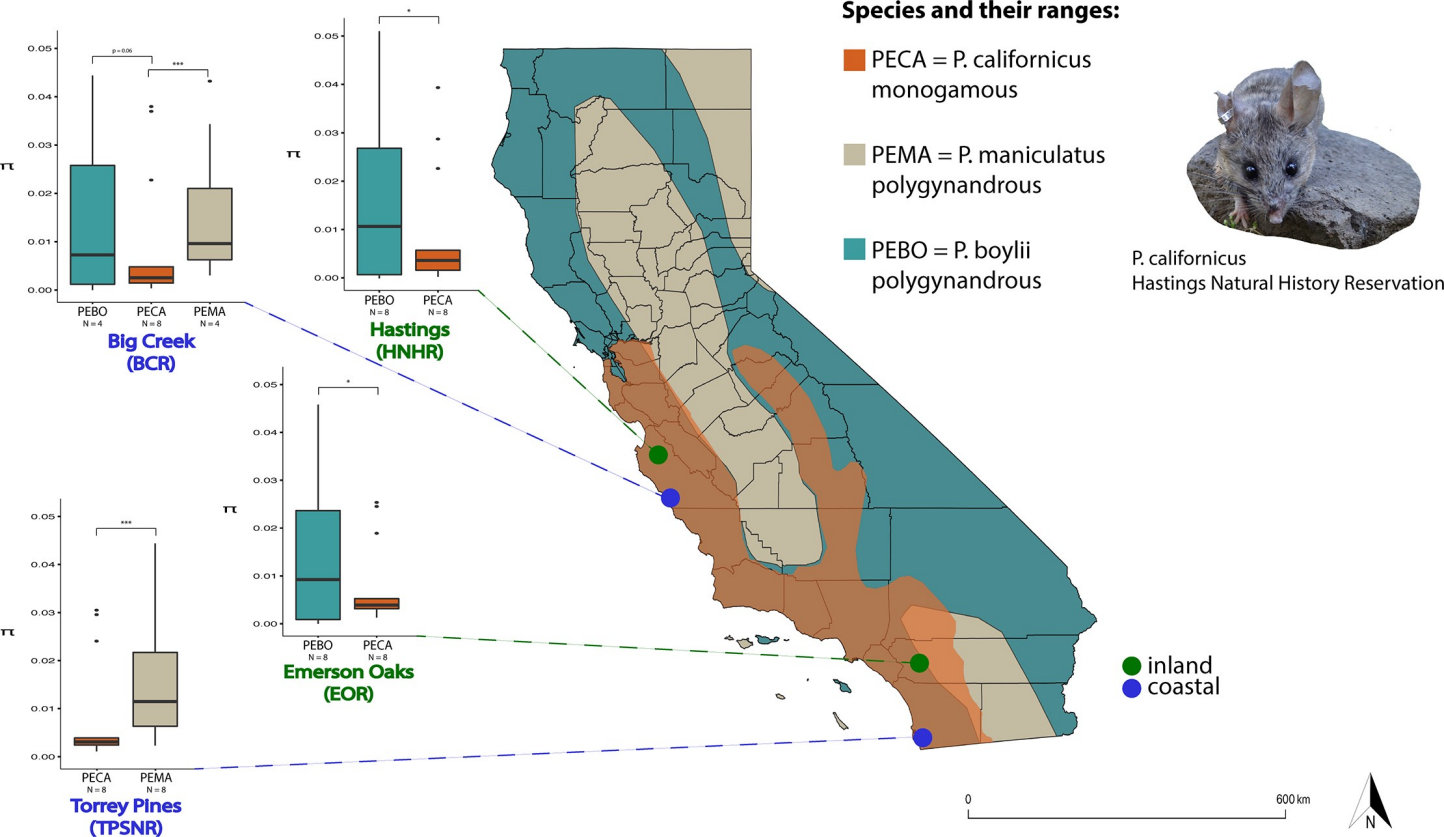
Field site localities in California and sampling regimes for the three species of *Peromyscus* examined; species abbreviations and geographic distributions are indicated. For each sampling locality, boxplots depicting population-level per-site nucleotide diversity (π) for SNPs associated with MHC loci (N = 13 loci per species per population) are shown, as is the number of adults sampled for RNAseq analyses. Northern (Big Creek and Hastings) and southern (Emerson Oaks and Torrey Pines) sampling localities are indicated; coastal (blue) versus inland (green) localities are identified.

Mice were sampled at four localities, two in the northern and two in the southern portions of the range of *P*. *californicus* (Fig 1). The two northern populations were located at the Landels-Hill Big Creek Reserve, Big Sur, CA (BCR) and the Hastings Natural History Reservation, Carmel Valley, CA (HNHR). The two southern populations were located at the Emerson Oaks Reserve, Temecula Valley, CA (EOR) and the Torrey Pines State Natural Reserve, La Jolla, CA (TPSNR). From north to south, the distribution of this species is characterized by a pronounced rainfall gradient, with southern populations generally experiencing more arid conditions (California precipitation maps: www.cnrfc.noaa.gov); inclusion of populations from both extremes of the range of *P*. *californicus* encompassed habitat variation associated with this marked difference in rainfall. Similarly, because habitats differ substantially on the western versus the eastern sides of the coastal mountains in California (1981–2010 Climate normals: www.cnrfc.noaa.gov), at each end of our north-south sampling transect we selected one coastal and one inland population for analysis, resulting in a total of four sampling localities. At both coastal locations, *P*. *californicus* co-occurs with *P*. *maniculatus*; at both inland locations, *P*. *californicus* co-occurs with *P*. *boylii*. Thus, this sampling scheme allowed us to compare genetic diversity in the monogamous species to that in two different polygynandrous species, with each interspecific comparison encompassing two distinct habitat types. This sampling scheme was not intended to provide a detailed analysis of the potential role of environmental differences on MHC variability in the study species but, rather, to assess the generality of relationships between MHC variation and mating system across differences in the conditions in which the study species occur.

#### Trapping and tissue collection

All trapping of the study populations was completed between February and April 2016. At each sampling locality, animals were captured using Sherman live-traps baited with rolled oats and containing a small ball of synthetic batting that the animals used as nesting material. A total of 180 traps were set per locality, with traps placed in pairs at 10 m intervals to create a grid measuring 150 m x 60 m and containing 90 trap stations (pairs of traps). At each sampling locality, traps were opened at 1600 hrs and closed 0300 hrs for 20 consecutive nights. All animals captured were identified to species using standard pelage and body size characters [21].

At each sampling locality, we collected a subset of seven adult males and seven adult females per species (total N = 112 animals) for use in transcriptomic analyses. These animals were euthanized via overdose with Isoflurane followed by cervical dislocation. Immediately postmortem, a sample of liver tissue (0.5 cm x 0.5 cm) was collected from each individual. Liver samples were placed in 1.5 mL of RNAlater for 24 hrs at 4° C, after which samples were frozen in liquid nitrogen. In addition, a sample of ear pinna was collected from each animal for use in mitochondrial sequence confirmation of species identity. Pinna samples (2 mm x 5 mm) were stored in 95% ethanol at ambient temperature until analysis. At the end of each field trip, samples were transported to the Museum of Vertebrate Zoology on the UC Berkeley campus; once on campus, the samples were stored in a -80°C freezer until analysis.

All field work involving live mice was approved by the Animal Care and Use Committee at the University of California, Berkeley, and was consistent with the Guidelines for the Use of Wild Mammals in Research published by the American Society of Mammalogists [24].

### Molecular work and analyses

#### Genetic confirmation of species identity

Because the study species can be difficult to distinguish based solely on external pelage characteristics, for the subset of animals collected for transcriptomic analyses, we confirmed species assignments made in the field using sequences from the mitochondrial *cytochrome b* (*cyt-b*) locus. Genomic DNA was isolated from ear pinna samples using a salt extraction protocol [25]. PCR amplification of the entire 1140-bp *cyt-b* locus was performed using primers MVZ 05 and MVZ 16 [26]. Our PCR master mix consisted of 25μL containing the following: 14.58μL of ddH_2_O, 2.75μL of 10x buffer (with MgCl_2_), 2.2μL of MgCl_2_ (50mM), 2μL of Betaine (5M), 1μL of BSA (10mg/ml) (Bovine Serum Albumin), 0.44μL of dNTPs (10mM), 0.44μL of each of the primers (10μM) (forward and reverse), 0.15μL of Taq polymerase (5,000U/ml) (New England Bio Labs), and 1μL of the DNA template. Amplification conditions consisted of an initial denaturation at 95°C for 4:00 min and 35 cycles of the following: denaturation at 95°C for 0:35 min, annealing at 49°C for 0:40 min, and extension at 72°C for 0:50 min. Our cycle sequencing mix consisted of 9μL reactions containing the following: 5.94μL of ddH_2_O, 1.43μL of the forward primer (MVZ 05), 1.98μL of the 5x Big Dye Buffer, 0.5μL of Big Dye, and 1μL of the PCR template. Sequences were edited and aligned using Geneious 7.1.7 [27], after which each sequence was subjected a BLAST search in Genbank and species identity assigned based on the top BLAST sequence match (sequence identity > 98%) for that sample.

#### Isolation of RNA

To quantify transcriptomic patterns of genetic diversity, we first isolated RNA from a subset of the liver samples collected during this study (four males and four females per species per sampling location), resulting in extracts from 64 individuals. For each sample, a 15 mg portion of liver was homogenized using a PowerLyzer^®^ 24 equipped with ceramic beads (MO BIO Laboratories, Inc.), after which RNA was extracted from the homogenized sample using the UltraClean^®^ Tissue and Cells RNA isolation kit (MO BIO Laboratories, Inc.). The concentration of each extract was determined using a NanoDrop spectrophotometer (ThermoFisher); typically, extractions yielded ~ 200 ng/μL of RNA. The quality of each extract was assessed using RNA 6000 Pico BioAnalyzer chips (Agilent); the mean RNA integrity number (RIN) score for our samples was 8 (range = 7.1 to 8.9). RNA extractions were then further purified using the DNase Max^®^ kit (MO BIO) or—for samples requiring additional concentration—RNeasy spin columns (Qiagen). Finally, samples were diluted to a standard concentration of 80 ng/μL in 25 μL of RNAase-free water (total = 2000 ng RNA) for library preparation.

#### Library preparation and transcriptomic sequencing

cDNA libraries were prepared using the Illumina^®^ platform KAPA Stranded mRNA-Seq Kit (KAPA Biosystems), with the manufacturer’s protocol modified to accommodate half reactions. For each sample, a total of 2μg of high-quality RNA (RIN > 7) was suspended in 25μL of RNase-free water. Samples were fragmented at 94°C for 4 min in order to achieve a typical library insert size of 250bp. We made two modifications to the manufacturer’s protocol. First, to minimize artifacts resulting from over-amplification and to maximize the number of unique fragments amplified, we divided the cleaned, adaptor ligated cDNA samples (step 10, KAPA technical data sheet) in to two amplification reactions. Each of these sub-reactions was amplified for 10 cycles, after which the reactions were pooled to produce a single final library per individual. Second, the final, uniquely barcoded libraries were cleaned using low ratio SeraMag beads (Sigma-Aldrich) instead of KAPA pure beads.

Initially, the size and concentration of each library were assessed visually by running samples on a 1% agarose gel that was then stained with ethidium bromide. More precise measures of library sizes were obtained using a BioAnalyzer DNA 1000 chip; mean library size (without primer sequences) was 264 (±13bp). More precise measures of library concentrations were obtained using a Qubit fluorometer (ThermoFisher) high sensitivity assay. Libraries measuring < 10ng/μL were further concentrated using a 2x Solid Phase Reversible Immobilization (SPRI) bead clean up procedure. A 10μL aliquot of each final library was submitted to the Vincent J. Coates Genomics Sequencing Laboratory at the University of California, Berkeley for sequencing. The 64 samples submitted were pooled equimolarly and then distributed across four lanes of Illumina HiSeq4000, 100PE sequencing.

#### Raw data processing

Raw sequence data were cleaned following the protocols of Singhal (2013) [28] and Bi et al., (2012) [29]. In brief, adaptor contamination was removed from the fastq reads using cutadapt [30], after which the remaining sequences were filtered by using Trimmomatic [31] to remove low quality reads (PHRED < 20). We then removed duplicate reads using Super-Deduper (https://github.com/dstreett/Super-Deduper). Finally, we identified reads resulting from bacterial contamination by aligning all sequences in our dataset to the *Escherichia coli* genome, after which contaminant reads were removed using Bowtie2 [32].

#### Construction of species-specific reference assemblies

To examine genetic diversity and patterns of selection on protein coding genes, we created species-specific coding sequence (CDS) reference assemblies for each of our study taxa. We began by downloading the existing CDS reference assembly for *P*. *maniculatus* available from the NCBI RefSeq database (GCF_000500345.1_Pman_1.0_cds_from_genomic.fna). Such CDS references typically contain multiple transcripts per gene; because we were interested in genic level patterns of diversity and selection, we used custom Biopython scripts [33] to create a CDS reference for *P*. *maniculatus* that contained only the single longest transcript variant for each gene in the assembly.

To increase the efficiency and accuracy of read mapping for the other study species and to reduce mapping bias caused by use of a taxonomically distinct reference, we constructed species-specific CDS references for *P*. *californicus* and *P*. *boylii*. We selected one male and one female per species per study population and then concatenated the reads for all conspecifics. We indexed the single-copy *P*. *maniculatus* CDS reference using the LAST aligner *lastdb* command, after which we used the LAST aligner *fastq-interleave* module to merge the concatenated sequences for *P*. *californicus* and *P*. *boylii* and to align the resulting reads for each species to the indexed single-copy CDS reference for *P*. *maniculatus*. Each aligned sequence was converted to BAM format using SAMtools [34], after which alignments were sorted using *samtools sort* and indexed using *samtools index*. These alignments were then converted to FASTA format CDS references for each species using the *samtools mpileup* and *seqtk seq* functions (https://github.com/lh3/seqtk); the resulting reference sequences were evaluated for completeness by comparing them to the single-copy *P*. *maniculatus* CDS reference. Missing data for *P*. *californicus* and *P*. *boylii* were replaced with the corresponding portion of the *P*. *maniculatus* reference. Sites denoted by IUPAC ambiguity codes were replaced by randomly selecting one of the possible nucleotides present at that location (e.g. inserting a C or a T for a nucleotide denoted as Y).

#### Identifying Single Nucleotide Polymorphisms (SNPs)

We used SNP markers to quantify genetic variation in each study species. To account for potential errors during base calling, alignment, or assembly, whenever possible we used genotype likelihoods instead of fixed genotypes to quantify genetic variability. Genotype likelihoods were calculated using the empirical Bayesian framework in ANGSD (http://www.popgen.dk/angsd/index.php/ANGSD) [35]. This program is particularly well suited to analyzing low to medium coverage genomic sequencing data because many of the analyses contained in this software package employ likelihood estimates or posterior probabilities to determine allele frequencies and genotypes. To increase the reliability of genotypic assignments for analyses conducted outside of ANGSD, we only considered sites for which at least 80% of samples were characterized by > 5x coverage. We identified genotypic variants using a likelihood ratio test; high confidence assignments had genotype posterior probabilities of > 0.95 and a p-value of < 1.00^−06^.

To examine relationships between mating behavior and immunogenetic diversity, we identified SNPs in our data set that were associated with MHC Class I and II loci based on gene nomenclature. We extracted genes that matched known naming systems for MHC (e.g. H-2, HLA, MHC, major histocompatibility). Similarly, we identified genes coding for MHC transactivator proteins and regulatory factors (S1 Table). Collectively, we refer to these genes as ‘MHC loci’. To provide a comparative data set of presumptively neutral loci, we used ‘BayeScan’ (pr_odds = 100) [36] to identify and then remove any outlier loci (genes potentially subject to divergent selection) from the remaining, non-MHC loci for each species; the loci retained after this analysis are referred to in the results as the “neutral” data set.

#### Genetic diversity and departures from neutrality

For all MHC loci as well as for all neutral loci in our data set, overall genetic variation was estimated in ANGSD as both the average number of pairwise differences, π [37], and the total number of segregating sites, Watterson’s θ [38]. Under equilibrium, average nucleotide differences per site (π) are not expected to differ from the number of segregating sites (θ), such that *π = 4N*_*e*_*μ = θ*. To test for possible deviations from this expectation, we used ANGSD to calculate Tajima's *D* [39] for each SNP locus included in our MHC data set. Additionally, we tested each locus for evidence of departures from Hardy-Weinberg equilibrium using the R package ‘pegas’ [40]. Taken together, these metrics allowed us to compare patterns of genetic diversity for MHC loci at both the population- and the species-level. Finally, to confirm that conspecifics sampled at the same locality were not close kin, we estimated coefficients of relatedness among individuals using the ‘SNPRelate’ package in R [41].

#### Tests for selection

To test the prediction that polygynandry is associated with enhanced selection on MHC loci, we quantified the apparent strength of selection on these genes in each of our study species. To begin, we reconstructed full sequences for our loci and used the KaKs_calculator [42] to test for nucleotide divergence in codon alignments for orthologous pairs of loci. For these analyses, we used the model averaging (MA) method, which employs a candidate model approach and maximum likelihood framework to select the best-fit model of nucleotide evolution. In addition to examining evidence for selection on all MHC loci in our data set, we also explored evidence for selection within the peptide-binding regions (PBR) of two MHC Class II loci that have been used in multiple previous studies of free-living vertebrates: the MHC Class II *DQα* and *DRβ* genes. Positive selection is only expected to operate within the peptide-binding regions (PBR) of Class II loci [43]; we were unable to identify exon 2 from a third commonly used Class II locus (*DQβ*) and thus tests for selection were not performed on this gene.

We used the branch-site model of diversifying selection as implemented in the *codeml* package in PAML [44,45] to test for positive selection on exon 2 of *DQα* and exon 2 of *DRβ* in each of our study species as well as to identify individual codons that appeared to be subject to positive selection. For these analyses, we first downloaded available exon 2 sequences for *P*. *californicus* MHC-*DQα* (Genbank JN703316) and *P*. *maniculatus* MHC-*DRβ* (NCBI AF516929.1). We then generated a consensus sequence for each locus and species (N = 6 sequences) in Clustal Omega [46]. We examined each consensus sequence for evidence of selection by contrasting a null model (no selection) for sequence variation against a model based on positive selection. For both models, we designated the species being tested as the ‘foreground’ branch in the underlying phylogenetic tree, with both other species designated as ‘background’ branches; each study species was used as the ‘foreground’ for each gene (total N = 6 comparisons). In the model of positive selection, the foreground branch was allowed to have a subset of sites evolving under positive selection (model = 2, fix_omega = 0, NSsites = 2). In contrast, for the null model, foreground branch sites were assumed to be evolving neutrally (model = 2, fix_omega = 1, NSsites = 2). The two models were compared using a log-likelihood ratio test in which the value of twice the difference between the log-likelihood of each model (*2ΔlnL = 2(lnL*_*1*_ *- lnL*_*0*_*)*) was compared to a *X*^*2*^ distribution with one degree of freedom.

#### Genetic differentiation among populations

To examine potential differences in genetic structure among the study populations, we used the ‘dartR’ [47] package in R to calculate the Weir and Cockerham (1984) estimator of F_ST_ for all pairwise combinations of conspecifics from different populations. Within species, we used this procedure to estimate F_ST_ for neutral loci in our data set as well as for the MHC genes examined. As described above, neutral loci excluded both MHC genes and outlier SNPs identified using ‘BayeScan' [36]. As an additional means of exploring the immunogenetic structure of our study populations, for each species we performed an Analysis of Molecular Variance (AMOVA; [48]) on SNPs associated with MHC loci as well as for all neutral SNPs examined. Geographic region (north or south) was the highest hierarchical level of organization for both *P*. *californicus* and *P*. *boylii*, and population was the highest level for *P*. *maniculatus*. To execute AMOVAs, we used the R packages ‘adegenet’ [49,50], ‘poppr’ [51], ‘pegas’ [40] and ‘ape’ [52]. Significance of the AMOVA output was assessed using a random permutation approach (1,000 permutations) as implemented in the ‘pegas’ R package [40]. Finally, we tested for correlations between genetic distance (Nei’s distance (1972)) [53] and geographic distance (kilometers) using a mantel test as implemented in the ‘vegan’ R package [54]. Significance of the mantel test was assessed using a random permutation approach (1,000 permutations) as implemented in the ‘vegan’ R package [54]. Mantel tests were only conducted for *P*. *californicus* and *P*. *boylii*; data from *P*. *maniculatus* were excluded from these analyses because samples for this species were only available from two localities (Fig 1). Finally, to determine if genetic structure differed between MHC and neutral loci within each study species, we used a co-inertia analysis as implemented in the ‘ade4’ R package [55], with the strength of the association between these subsets of genes evaluated using a Monte Carlo permutation (N = 99 iterations).

## Results

### Molecular confirmation of species identity

For the 64 animals used in the RNAseq experiment, analyses of *cyt-b* sequences confirmed that all individuals identified in the field as *P*. *californicus* (n = 32) and *P*. *boylii* (n = 16) were correctly assigned to these species. Of the 16 animals identified in the field as *P*. *maniculatus*, two males and two females from the northern, coastal BCR population were identified genetically as *P*. *boylii*. As a result, the final sample size for each species was 32 for *P*. *californicus*, 20 for *P*. *boylii*, and 12 for *P*. *maniculatus;* the final data set for the BCR population included data from all three study species (Fig 1).

### RNA sequencing analyses

Each of our four Illimuna HiSeq4000 lanes yielded ~ 390 M reads, resulting in a total of ~ 1,545 M reads. No samples failed our quality control thresholds and thus all reads were retained for subsequent analyses. Across all four lanes of sequence, approximately 93% of nucleotides had Q scores > 30 (range = 87–95%), indicating that the error rate for reading nucleotides was < 0.1%. Average coverage for the 64 individuals sequenced was 42% (± 7%).

### Identification of Single Nucleotide Polymorphisms (SNPs)

A total of 125,720 SNPs were identified for *P*. *californicus*, 247,602 for *P*. *maniculatus*, and 53,022 for *P*. *boylii*. Of these, the number associated with MHC genes was 988 for *P*. *californicus*, 1,847 for *P*. *maniculatus* and 1,712 for *P*. *boylii*. The number of distinct MHC loci represented per study species was 28, 29 and 33, respectively; overall, 89% of the MHC genes represented in the SNP data set were shared across all three species.

### Patterns of genetic diversity

**Fig 2:**
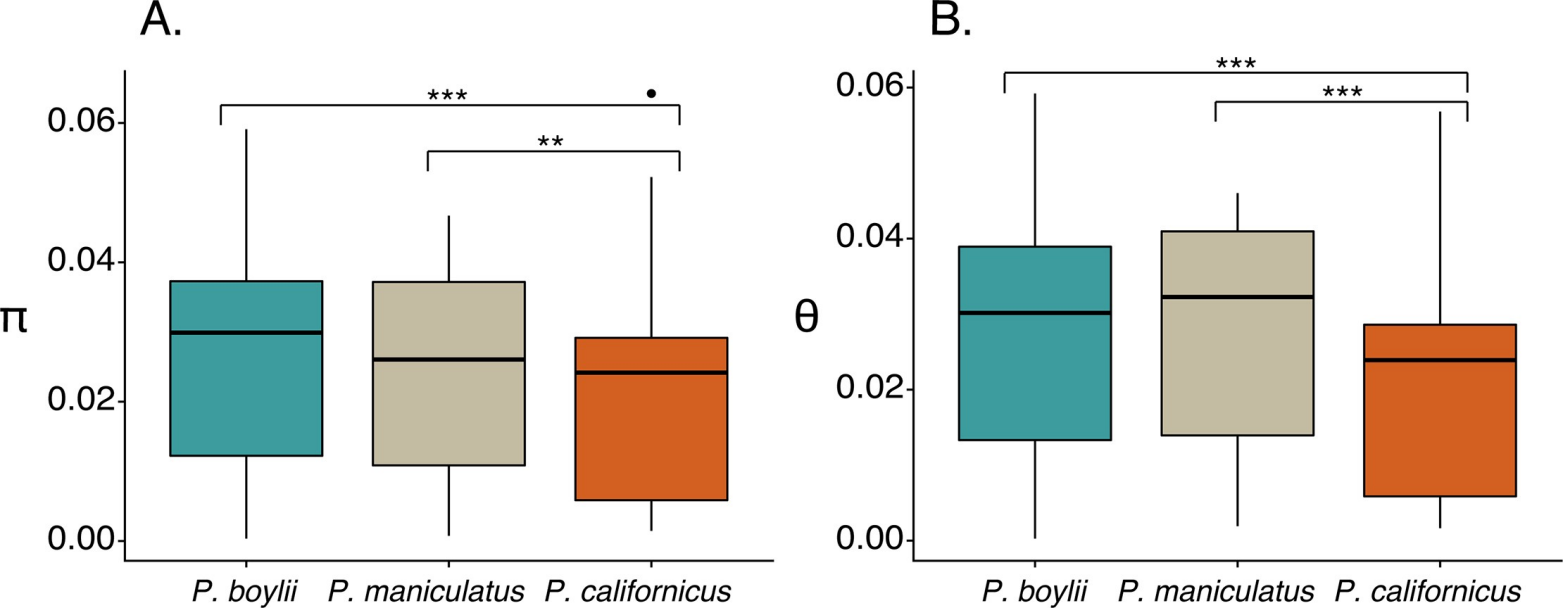
Species-level estimates of diversity for MHC genes. Species-level estimates of (A) per-site nucleotide diversity (π) and (B) number of segregating sites (θ) for SNPs associated with 27 MHC genes identified in our study species. Estimates of π and θ for each species were calculated by pooling data from all populations of conspecifics. For each species, box plots denote median values of these estimators. Number of individuals sampled for each species are as follows: P. boylii (N = 20), P. maniculatus (N = 12), P. californicus (N = 32). Significant contrasts (Wilcoxon Signed Rank tests, p < 0.05) are indicated with brackets and asterisks.

Between-species comparisons of genetic diversity were restricted to genes detected in all three species; this restriction was applied to analyses of MHC loci as well as analyses of all SNPs examined (Table 1). At the species level, average values of π and θ for MHC loci differed significantly among the study taxa (Friedman rank sum test, all p < 0.01) (Table 1 and Fig 2). Post-hoc tests indicated that for both parameters (π and θ), values for the two polygynandrous species were greater than those for the monogamous species (Wilcoxon signed rank tests, all p < 0.01, Bonferroni corrected alpha = 0.017) but that there were no significant differences between the polygynandrous species (Wilcoxon signed rank test, all p > 0.01).

**Table 1:** Genetic diversity in three species of *Peromyscus*. Analyses were conducted for (A) SNPs associated with a subset of MHC loci shared among species/populations and (B) all SNP loci shared among species/populations; the number of genes included in each data set is given in parenthesis under population names.

A. MHC loci										
Species	All populations (N = 27)		BCR (N = 13)		HNHR (N = 13)		EOR (N = 13)		TPSNR (N = 13)	
	π	Θ	π	θ	π	θ	π	θ	π	θ
*P*. *californicus*	2.2^−02^ ±0.02	2.0^−02^ ±0.01	9.3^−03^ ±0.01	8.1^−03^ ±0.01	9.1^−03^ ±0.01	7.4^−03^ ±0.01	7.9^−03^ ±0.01	7.5^−03^ ±0.01	8.6^−03^ ±0.01	7.4^−03^ ±0.01
*P*. *boylii*	2.7^−02^ ±0.02	2.7^−02^ ±0.02	1.4^−02^ ±0.01	1.2^−02^ ±0.01	1.6^−02^ ±0.02	1.3^−02^ ±0.01	1.5^−02^ ±0.02	1.4^−02^ ±0.02	n/a	n/a
*P*. *maniculatus*	2.5^−02^ ±0.01	2.8^−02^ ±0.02	1.5^−02^ ±0.01	1.5^−02^ ±0.01	n/a	n/a	n/a	n/a	1.6^−02^ ±0.01	1.6^−02^ ±0.01
B. Neutral loci										
Species	All populations (N = 10,023)		BCR (N = 17,113)		HNHR (N = 17,369)		EOR (N = 17,395)		TPSNR (N = 17,544)	
	π	Θ	π	θ	π	θ	π	θ	π	θ
*P*. *californicus*	2.4^−03^ ±0.003	2.7^−03^ ±0.003	2.2^−03^ ±0.003	2.1^−03^ ±0.002	2.0^−03^ ±0.003	1.9^−03^ ±0.002	2.7^−03^ ±0.003	2.7^−03^ ±0.002	2.6^−03^ ±0.003	2.5^−03^ ±0.002
*P*. *boylii*	1.2^−03^ ±0.003	1.1^−03^ ±0.003	1.3^−03^ ±0.003	1.1^−03^ ±0.002	1.4^−03^ ±0.003	1.1^−03^ ±0.002	1.7^−03^ ±0.003	1.5^−03^ ±0.003	n/a	n/a
*P*. *maniculatus*	4.7^−03^ ±0.004	5.4^−03^ ±0.004	6.4^−03^ ±0.004	6.7^−03^ ±0.004	n/a	n/a	n/a	n/a	6.0^−03^ ±0.004	6.1^−03^ ±0.004

For each set of loci, mean (± SD) values of per-site nucleotide diversity (π) and number of segregating sites (θ) were calculated; data from each species were analyzed with all populations of conspecifics pooled (‘All populations’; a species-level estimate) and with each population examined separately (a population-level estimate) (population abbreviations explained in Fig 1).

At the population level, measures of genetic diversity also tended to be greater for the polygynandrous study species. For the two localities at which P. maniculatus and P. californicus co-occurred (Fig 1 and Table 1), populations of the former were characterized by significantly greater diversity at MHC genes (Wilcoxon signed rank tests, all p < 0.001). For the three localities at which P. boylii and P. californicus co-occurred, average values of π and θ for MHC genes were typically greater in P. boylii (Wilcoxon signed rank tests, all p < 0.05); the sole exception was the BCR population, in which average π did not differ between these species (Wilcoxon signed rank test, p = 0.06) (Fig 1 and Table 1). Within species, no significant differences in average π and θ for MHC genes were detected among populations (Friedman rank sum tests, all p > 0.05).

**Fig 3:**
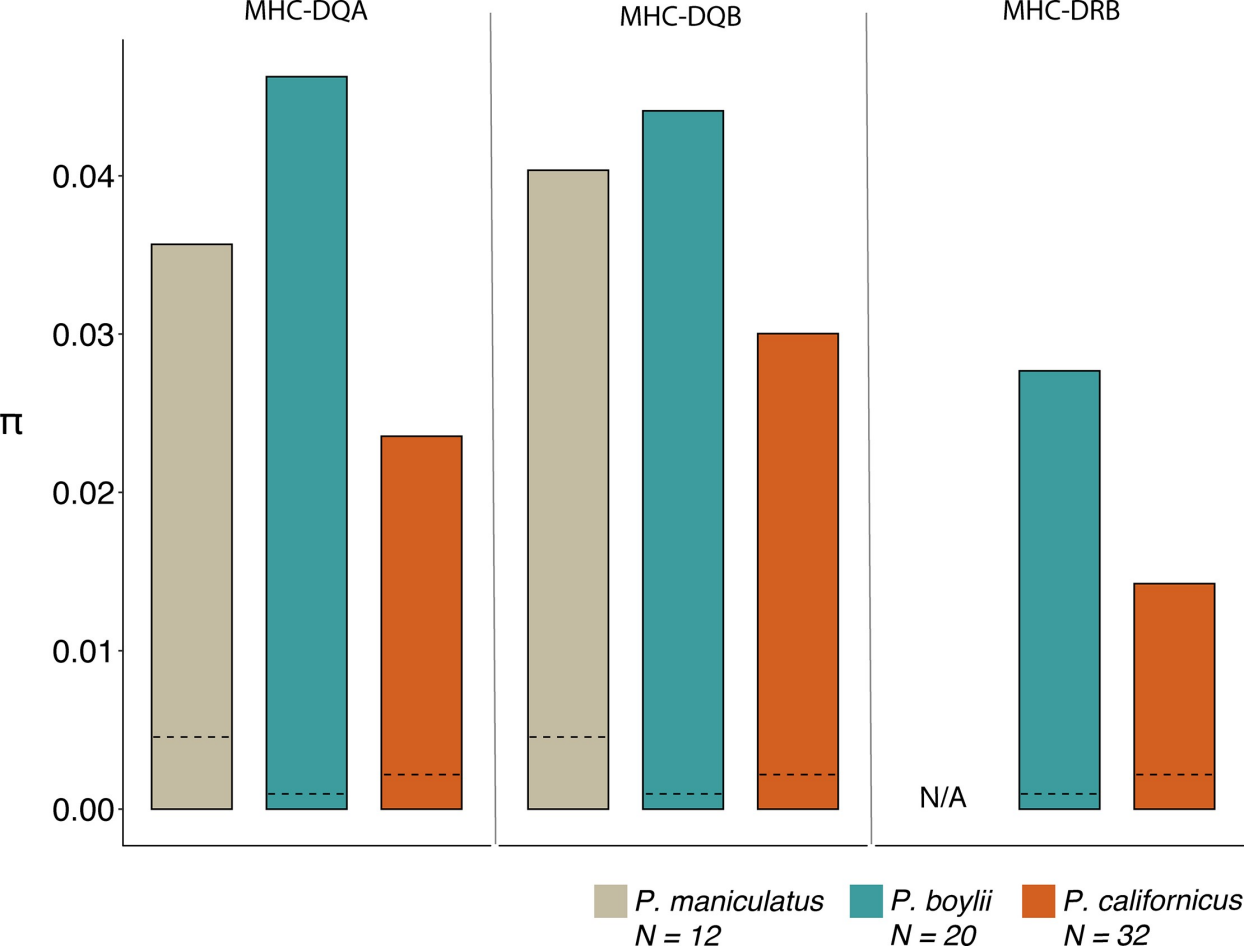
Species-level estimates of per-site nucleotide diversity (π) for 3 commonly examined Class II MHC loci. For each locus, estimates of π for each species were calculated by pooling data from all populations of conspecifics. The number of individuals sampled per species is shown in the legend. The dashed lines within bars indicate overall genetic diversity as estimated from all SNP loci examined for each species. No variation was detected at the *DRB* locus in *P*. *maniculatus* and thus π for this locus could not be estimated for this species.

At the level of individual MHC loci, species-level comparisons of diversity revealed that for the MHC Class II *DQα* and *DQβ* genes, values of π were greater in *P*. *boylii* and *P*. *maniculatus* than in *P*. *californicus* (Fig 3). Estimates of π for the *DRβ* locus were also greater for *P*. *boylii* than for *P*. *californicus*; our analyses failed to reveal any variable sites in this gene in *P*. *maniculatus* and thus we could not estimate π for this species. Thus, for each of the individual MHC genes examined, there was a tendency for both measures of diversity to be reduced in the monogamous compared to the polygynandrous study species.

### Estimates of genetic relatedness

Coefficients of relatedness calculated based on data from all SNPs revealed that in *P*. *californicus*, overall pairwise relatedness among members of the same population tended to be low (mean *r* = 0.004 ± 0.04). Coefficients of relatedness calculated using only data from MHC loci were also low (mean *r* = 0.02 ± 0.07). Similarly, pairwise coefficients of relatedness for *P*. *maniculatus* tended to be low, with mean values of 0.01 ± 0.07 (all loci) and 0.10 ± 0.25 (MHC loci). Pairwise estimates of relatedness for *P*. *boylii* were comparable, with mean values of 0.02 ± 0.05 (all loci), and 0.04 ± 0.10 (MHC loci). Thus, overall, conspecifics sampled at the same locality were not closely related to one another.

### Departures from neutrality

Overall, distributions of Tajima’s *D* for MHC loci approximated normality (Shapiro-Francia neutrality tests, all p < 0.03) and thus we expected ~ 95% of locus-specific values of *D* to fall between –2 and 2. The average (± SD) value of Tajima’s *D* in the BCR population of *P*. *californicus* was weakly negative (-0.05 ± 0.96) but was weakly positive in the remaining three populations of this species (HNHR: 0.42 ± 0.99; EOR; 0.12 ± 0.47; TPSNR; 0.34 ± 0.62); no loci displayed significant departures from neutral expectations (all p > 0.05). In *P*. *maniculatus*, values of Tajima’s D were also weakly negative in the BCR population (-0.36 ± 0.80) but weakly positive in the TPSNR population (0.04 ± 0.59), with no departures from neutral expectations (all p > 0.05). In contrast, in *P*. *boylii*, average values of D were weakly positive in all populations (BCR: 0.71 ± 0.40; HNHR: 0.77 ± 0.91; EOR: 0.96 ± 1.96), with several genes displaying apparent departures from neutrality at EOR and HNHR (*D* > 2 or *D* < -2) (Table 2). With the exception of the MHC Class II transactivator protein (*Ciita*) locus, significantly positive values of Tajima’s D were obtained for MHC genes (Table 2).

**Table 2:** MHC loci displaying departures from neutral expectations as determined from values of Tajima’s *D*. All loci displaying such departures were detected in *P*. *boylii*.

Gene ID	Gene Name	Tajima's D	Population	potential selective force
XM_015991210.1	patr class I histocompatibility antigen, A-126 alpha chain-like	3.45	EOR	balancing
XM_015989456.1	popy Class I histocompatibility antigen, A-1 alpha chain-like	2.66	EOR	balancing
XM_015990243.1	HLA class II histocompatibility antigen, DO beta chain	2.43	EOR	balancing
XM_006996070.1	saoe class I histocompatibility antigen, A alpha chain-like	2.70	EOR	balancing
XM_006997045.2	class II histocompatibility antigen, M alpha chain	2.65	EOR	balancing
XM_016002443.1	class II, major histocompatibility complex, transactivator (Ciita)	-3.79	EOR	purifying
XM_015991210.1	patr class I histocompatibility antigen, A-126 alpha chain-like	2.35	HNHR	balancing

Multiple SNPs associated with MHC genes displayed evidence of departures from Hardy-Weinberg expectations. Across populations of *P*. *californicus*, an average of 64% (± 7%) of the SNPs examined (N = 988) differed significantly from Hardy-Weinberg expectations. This value was 85% (± 5%) (N = 1,847) for *P*. *maniculatus* and 76% (± 5%; N = 1,712) for *P*. *boylii*. When we examined the specific MHC genes that contained these SNPs, we found that ~ 70% of these loci were shared among data sets for all three of the study species. In contrast, two of these genes were detected only in *P*. *californicus* (HLA class I histocompatibility antigen, A-36 alpha chain-like; H-2 class I histocompatibility antigen, Q9 alpha chain-like), one was detected only in *P*. *maniculatus* (patr class I histocompatibility antigen, A-126 alpha chain-like), and two were detected only in *P*. *boylii* (H-2 class II histocompatibility antigen, E-D beta chain-like; H-2 class I histocompatibility antigen, alpha chain-like). Thus, the vast majority of SNPs that were characterized by significant departures from neutrality were present in the monogamous as well as the polygynandrous study species.

### Tests for selection

**Fig 4:**
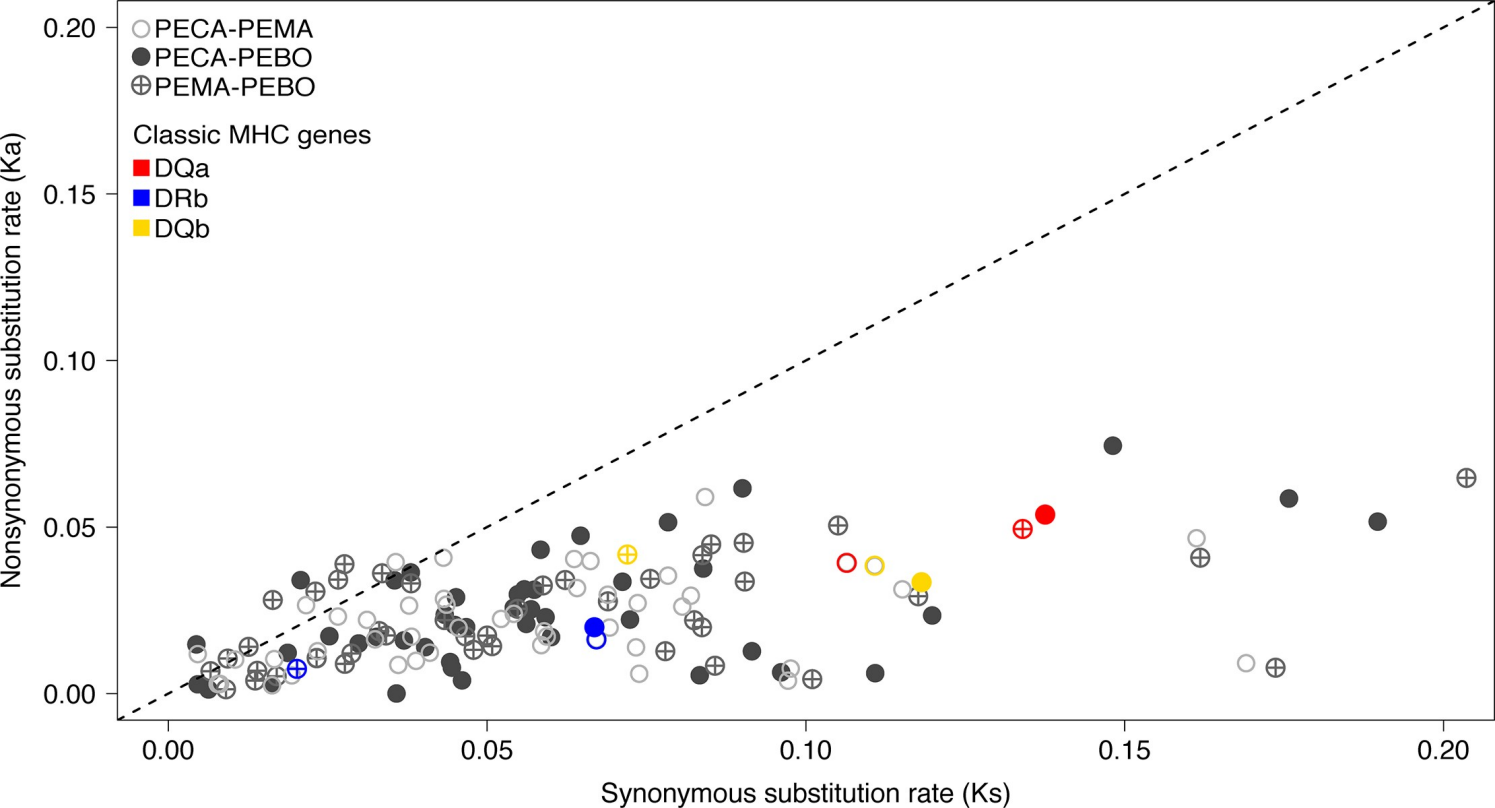
Distribution of Ka/Ks ratios for SNPs associated with orthologous pairs of MHC genes. Each value represents a pairwise comparison between two of the study species; the symbols used to denote each species pair are shown in the legend (PEBO = *P*. *boylii*; PEMA = *P*. *maniculatus*; PECA = P. *californicus*). Data from three commonly studied MHC genes are shown in color; the color used to denote each locus is also shown in the legend. The dashed line indicates a Ka/Ks ratio of 1; points falling above this line indicate an excess of non-synonymous substitutions, as expected for loci subject to positive selection.

In general, the MHC genes that displayed significant evidence of selection appeared to be subject to purifying selection (Fig 4). Of the three classic MHC genes examined (*DQα*, *DRβ*, and *DQβ*), the Class II *DQα* locus appeared to be under purifying selection in all three species (p < 0.001 Fishers exact test). In contrast, rates of synonymous substitutions at the Class II *DRβ* and *DQβ* genes were greater for *P*. *maniculatus* and *P*. *boylii* than for *P*. *californicus*, with evidence for purifying selection detected only for *P*. *californicus* (PECA: p < 0.001; PEMA and PEBO: p > 0.05, Fishers exact tests). Branch-site tests for the peptide-binding regions (exon 2) of the *DQα* and *DRβ* genes revealed no evidence of positive selection in any of the study species (log-likelihood ratio test, all p > 0.05).

### Genetic differentiation among populations

Estimates of F_ST_ based on neutral loci as well as those based only on MHC genes indicated that genetic dissimilarity tended to increase with the geographic distance between populations (Tables 3 and 4). In *P*. *californicus*, the degree of differentiation among populations varied with gene category, with differentiation relative to geographic distance being greater for neutral loci than for MHC loci only. Differences between these data sets were less evident for the other study species and, overall, populations of *P*. *maniculatus* and *P*. *boylii* were characterized by smaller pair-wise estimates of F_ST_, suggesting that populations of these species were genetically less differentiated in relation to geographic distance than populations of *P*. *californicus*.

**Table 3:** Pairwise estimates of F_ST_ for populations of three species of *Peromyscus*.

Species	Pairwise site comparison	F_ST_	Location comparison
*P*. *californicus* (N = 988 SNPs)	EOR-BCR	0.13	North-South
	HNHR-BCR	0.02	North-North
	TPSNR-BCR	0.10	North-South
	HNHR-EOR	0.11	North-South
	TPSNR-EOR	0.03	South-South
	TPSNR-HNHR	0.08	North-South
*P*. *maniculatus* (N = 1,847 SNPs)	TPSNR-BCR	0.09	North-South
*P*. *boylii* (N = 1,712 SNPs)	EOR-BCR	0.13	North-South
	HNHR-BCR	0.05	North-North
	HNHR-EOR	0.10	North-South

Data are from all SNPs associated with MHC loci that were identified for each species; the number of loci analyzed is given below each species name. The relative geographic location of each population is indicated. No values of F_ST_ exceeded the threshold (0.15) for moderate genetic differentiation among populations (Hartl and Clark 1997).

**Table 4:** Pairwise estimates of F_ST_ for populations of three species of *Peromyscus*.

Species	Pairwise site comparison	F_ST_	Location comparison
*P*. *californicus* (N = 124,733 SNPs)	EOR-BCR	**0.42**	North-South
	HNHR-BCR	0.02	North-North
	TPSNR-BCR	**0.44**	North-South
	HNHR-EOR	**0.42**	North-South
	TPSNR-EOR	0.06	South-South
	TPSNR-HNHR	**0.44**	North-South
*P*. *maniculatus* (N = 245,756 SNPs)	TPSNR-BCR	0.09	North-South
*P*. *boylii* (N = 51,190 SNPs)	EOR-BCR	0.14	North-South
	HNHR-BCR	0.08	North-North
	HNHR-EOR	0.14	North-South

Data are from neutral SNPs identified for each species; the number of loci analyzed is given below each species name. The relative geographic location of each population is indicated. Values of F_ST_ denoting great (0.15–0.25) or very great (> 0.25) genetic differentiation among populations are indicated in bold (Hartl and Clark 1997).

For each study species, results of AMOVAs for neutral loci as well as those for MHC loci revealed that the majority of genetic variation was contained within populations. Consistent with our estimates of F_ST_, genetic differentiation of neutral loci between regions (north versus south) was substantial, although not statistically significant (p > 0.05; Table 5). For MHC loci, genetic differentiation between regions (north versus south) was an order of magnitude lower and was not statistically significant (p > 0.05) (Table 6). For both the MHC and neutral loci, differentiation between populations of conspecifics within regions was significant (p < 0.05) for *P*. *californicus* and *P*. *boylii*. For these two species, Mantel tests revealed positive correlations between genetic distance (Nei’s) and geographic distance (km) for both MHC loci (*P*. *californicus* r = 0.2673, p < 0.001; *P*. *boylii* r = 0.2446, p = 0.002) and neutral loci (*P*. *californicus* r = 0.2929, p < 0.001; *P*. *boylii* r = 0.5691, p < 0.001). Co-inertia analyses revealed that variability in the MHC and neutral data sets were highly correlated (RV coefficients: *P*. *californicus* = 0.81, p < 0.01; *P*. *boylii* = 0.82, p < 0.01; *P*. *maniculatus* = 0.82, p < 0.01).

**Table 5:** Results of Analyses of Molecular Variance (AMOVA) for three species of *Peromyscus*.

***P*. *californicus***							
**ALL loci** (N = 125,720 SNPs)							
	Df	SSD	MSD	Sigma^2^	Variance %	Φ-statistic	p-value
Among regions (North and South)	1	191217.09	191217.09	10502.93	35.31	ϕ_CT_ 0.353	0.32
Among populations/regions	2	46340.56	23170.28	561.54	1.89	ϕ_SC_ 0.029	0.0040*
Within populations	28	522982.38	18677.94	18677.94	62.80	ϕ_ST_ 0.372	-
Total	31	760540.03	24533.55	29742.41	100		
***P*. *maniculatus***							
**ALL loci** (N = 247,602 SNPs)							
	Df	SSD	MSD	Sigma^2^	Variance %	Φ-statistic	p-value
Among populations	1	70194.67	70194.67	5413.5	11.58	ϕ_CT_ 0.116	0.001*
Within populations	10	413229.00	41322.90	41322.9	88.42	-	-
Total	11	483423.67	43947.61	46736.36	100		
***P*. *boylii***							
**ALL loci** (N = 53,022 SNPs)							
	Df	SSD	MSD	Sigma^2^	Variance %	Φ-statistic	p-value
Among regions (North and South)	1	24711.83	24711.83	1086.29	10.68	ϕ_CT_ 0.103	0.34
Among populations/regions	1	12567.19	12567.19	804.48	7.91	ϕ_SC_ 0.089	0.0000*
Within populations	17	140702.81	8276.64	8276.64	81.40	ϕ_ST_ 0.183	-
Total	19	177981.83	9367.47	10167.41	100		

All neutral SNPs identified in these species were examined; the number of loci analyzed for each species is indicated below. The numbers of individuals analyzed were 32 for *P*. *californicus* (N = 4 populations), 12 for *P*. *maniculatus* (N = 2 populations), and 29 for *P*. *boylii* (N = 3 populations). The significance of AMOVA outputs was assessed using Monte-Carlo tests. Df = Degrees of freedom; SSD = sum of the squares of the deviations; MSD = mean signed difference.

**Table 6:** Results of Analyses of Molecular Variance (AMOVA) for three species of *Peromyscus*.

***P*. *californicus***							
**MHC loci** (N = 988 SNPs)							
	Df	SSD	MSD	Sigma^2^	Variance %	Φ-statistic	p-value
Among regions (North and South)	1	566.23	566.23	20.38	9.42	ϕ_CT_ 0.094	0.32
Among populations/regions	2	480.34	240.17	6.312	2.92	ϕ_SC_ 0.032	0.034*
Within populations	28	5310.94	189.68	189.68	87.66	ϕ_ST_ 0.123	-
Total	31	6357.52	205.08	216.37	100		
***P*. *maniculatus***							
**MHC loci** (N = 1,847 SNPs)							
	Df	SSD	MSD	Sigma^2^	Variance %	Φ-statistic	p-value
Among populations	1	591.17	591.17	40.98	9.91	ϕ_CT_ 0.099	0.01*
Within populations	10	3726.13	372.61	372.61	90.09	-	-
Total	11	4317.29	392.48	413.59	100		
***P*. *boylii***							
**MHC loci** (N = 1,712 SNPs)							
	Df	SSD	MSD	Sigma^2^	Variance %	Φ-statistic	p-value
Among regions (North and South)	1	747.58	747.53	19.63	4.92	ϕ_CT_ 0.049	0.35
Among populations/regions	1	499.71	499.71	27.84	6.98	ϕ_SC_ 0.073	0.058
Within populations	17	5971.31	351.25	351.25	88.09	ϕ_ST_ 0.119	-
Total	19	7218.60	379.93	398.72	100		

Data from SNPs associated with MHC loci; the number of SNPs examined for each species is indicated below. The numbers of individuals analyzed were 32 *P*. *californicus* (N = 4 populations), 12 *P*. *maniculatus*, (N = 2 populations) and 29 *P*. *boylii* (N = 3 populations). The significance of AMOVA outputs was assessed using Monte-Carlo tests. Df = Degrees of freedom; SSD = sum of the squares of the deviations; MSD = mean signed difference.

## Discussion

Differences in immunogenetic diversity among our study species were consistent with predictions based on the mating systems of these animals, with the polygynandrous *P*. *maniculatus* and *P*. *boylii* displaying greater diversity at MHC genes than the monogamous *P*. *californicus*. Despite these differences in diversity, we found no evidence for enhanced selection at MHC loci in either of the polygynandrous species relative to the monogamous species. Further, we found no evidence to suggest that variation in habitat conditions contributed to patterns of immunogenetic variation among populations or species. Because our findings are based on analyses of more than a single population per species and represent animals sampled from diverse habitats and geographic regions, these data imply that mating systems play an important role in shaping patterns of immunogenetic diversity in these mammals.

### Predictors of immunogenetic variability

Although differences in mating systems provide a logical explanation for the interspecific differences in immunogenetic diversity reported here, other factors may have contributed to this outcome, notably differences in the underlying genetic structures of the study species as well as local differences in the communities of pathogens to which the study animals were exposed. Within some populations, overall genetic diversity was greater for the two polygynandrous species and this tendency may have influenced variability at the subset of immunogenetic loci examined. This possibility is particularly relevant for animals sampled at EOR and TPSNR, where estimates of genetic diversity for all SNP loci were also greater for the two polygynandrous species than for the monogamous species (S1 Fig). Our analyses, however, failed to reveal consistent evidence of selection on MHC loci, making it challenging to distinguish the effects of selection due to differences in mating systems from those of interspecific differences in overall genetic diversity.

Interspecific differences in the spatial distribution of genetic variation may arise due to demographic differences that affect processes such as gene flow and genetic drift [56–59]. For example, potential differences in dispersal between members of monogamous and polygynandrous species could lead to increased isolation of populations for one mating system relative to the other, with consequences for within-population variation in monogamous versus polygynandrous taxa. Spatial patterns of genetic diversity, however, did not differ markedly among our study species and were not correlated with sampling location. For both neutral and MHC SNP loci, genetic differentiation (F_ST_) among populations of conspecifics tended to increase with geographic distance in each species. For neutral loci, although populations of *P*. *maniculatus* and *P*. *boylii* were typically less genetically distinct than those of *P*. *californicus*, estimates of F_ST_ for all species fell within values typically interpreted as evidence of moderate differentiation, providing no suggestion of pronounced interspecific variation in the spatial structure of genetic diversity. Consistent with this, the results of AMOVAs indicated that within each study species, population differentiation increased with geographic distance, a pattern that was confirmed using Mantel tests. Finally, co-inertia analyses revealed that variability at MHC and neutral loci were highly correlated in each species, providing no indication that genetic structure differed between these two categories of loci. Collectively, these results suggest that in our study animals it is geographic distance–not habitat or location–that underlies genetic differentiation at both MHC loci and neutral loci. Although this outcome appears to be robust, it is possible that our sampling scheme, in particular our sampling of just one population per geographic region (north versus south, coastal versus inland) precluded detection of fine scale variation differences in genetic structure among the study populations that contribute to the differences in MHC variability reported here. Future studies that include additional populations of each species from a greater variety of habitats should further elucidate the roles of distance and environment in contributing to the patterns of immunogenetic variability reported here.

At the same time, because MHC class I and II genes are thought to be subject to selection imposed by pathogens [3,4], variation in the pathogen communities to which members of our study populations were exposed may also have contributed to the differences in MHC variability detected. Habitat type (e.g., humid coastal habitats versus more arid inland habitats) is expected to affect pathogen exposure due to variation in the optimal conditions under which different pathogens proliferate [56][51]. Although we did not assess pathogen exposure quantitatively, the distinct habitats represented by our four sampling localities suggest that variation in local pathogen communities was possible. While we cannot exclude the possibility that habitat-related differences in pathogen exposure influenced MHC variation in our study populations, any effects of such differences in exposure did not alter the consistent tendency for MHC variability to be greater in our monogamous study species. Interactions between environmental conditions, pathogen exposure, and variability at MHC genes are likely to be complex and temporally dynamic [5,60] and thus future studies should compare these aspects of the biology of our study species in greater detail.

### Role of selection on immunogenes

When analyses were restricted to MHC loci, the degree of genetic differentiation among populations of conspecifics was reduced, with this change in outcomes being most evident for *P*. *californicus*. This decrease in spatial differentiation for immune loci may reflect the effects of balancing selection on MHC genes [4]; such selection should serve to enhance diversity at MHC loci, leading to potentially greater sharing of alleles and thus reduced genetic differentiation among populations of conspecifics [61–63]. Previous, locus-specific studies of *P*. *maniculatus* reported a similar pattern of reduced genetic differentiation at MHC relative to neutral genes [64]. Although our estimates of Tajima’s D suggested balancing selection at several MHC genes in *P*. *boylii*, these analyses failed to detect similar signatures of selection in *P*. *californicus* or *P*. *maniculatus* and, overall, evidence for selection on MHC genes was inconsistent. For example, while Ka/Ks ratio tests for selection revealed that MHC loci were typically subject to purifying selection (as expected given rigorous functional constraints on MHC genes [43]), our analyses of individual Class II MHC genes failed to reveal evidence of selection on the functionally important protein binding regions of these loci. While small samples sizes may have limited our ability to detect selection on the immunogenes in our data set, ours is not the only study to fail to detect evidence of selection on MHC loci in free-living mammals. For example, locus-specific analyses of cotton rats (*Sigmodon hispidus*: [65]) and wood mice (*Apodemus sylvaticus*: [66]) also failed to reveal significant evidence of selection on Class II MHC genes. Thus, our findings add to a growing body of literature suggesting that selection on these loci may be more variable or more complex (leading to greater difficulty in detecting signatures of selection) than has typically been assumed, particularly among members of natural populations of vertebrates [67,68].

### Mating systems and MHC diversity

Although the mechanisms by which individual-level decisions (mate choice) and population-level dynamics (pathogen-mediated selection) are expected to affect variation at MHC genes are generally understood, interactions at the interface between individuals and populations have not been as well-characterized. Mating systems provide a logical target for exploring this intersection, as they reflect both individual reproductive decisions and population level patterns of exposure to selective agents such as socially transmitted pathogens. Our finding that patterns of immunogenetic diversity differed with mating system underscores the potential for such analyses to generate new insights into the processes mediating individual- versus population-level impacts on genetic variability. For example, the lower variability at MHC genes reported here for *P*. *californicus* versus *P*. *maniculatus* and *P*. *boylii* is consistent with reduced pathogen exposure due to the limited number of reproductive partners per individual in the monogamous species [69]. At the scale of individual mate choice decisions, reduced immunogenetic diversity may limit the utility of MHC genes as bases for mate choice despite the expectation that mate choice has greater potential impacts on reproductive success in monogamous species [70]. Under these circumstances, selection on MHC genes due to mate choice may be limited, which may contribute to the lack of disassortative mating at MHC loci reported for multiple monogamous species including *P*. *californicus* [71], the Malagasy giant rat (*Hypogeomys antimena*: [72]), grey wolves (*Canis lupus*: [73]), and Magellanic penguins (*Spheniscus magellanicus*: [74]). Comparative studies that evaluate relationships among mate choice decisions, pathogen exposure, and immunogenetic variation in the context of differences in mating system should prove valuable in clarifying how individual level patterns of behavior contribute to population level patterns of genetic diversity.

Previous analyses of MHC diversity in *Peromyscus* typically examined allelic or nucleotide level variation at one to a few MHC genes [9,75,76]. Particularly relevant is the work by MacManes and Lacey (2012), who compared variation at the MHC Class II *DQα* locus in *P*. *californicus* and *P*. *maniculatus* from the Big Creek Reserve (BCR), one of the sampling sites examined here. Their analyses revealed greater allelic and nucleotide level variation, as well as greater evidence for selection, in the polygynandrous *P*. *maniculatus*. Bacterial diversity in vaginal swabs collected from female mice was also greater for *P*. *maniculatus* than for *P*. *californicus* [69], leading MacManes and Lacey (2012) to conclude that the distinct mating systems of these species were associated with differences in exposure to sexually transmitted pathogens that, in turn, have contributed to enhanced variability at the *DQα* locus in the polygynandrous *P*. *maniculatus*. While our results regarding overall patterns of MHC diversity are consistent with this conclusion, we did not find evidence for enhanced selection at the *DQα* gene nor any of the other individual MHC loci examined. Potential explanations for this disparity include differences in sample size, in the relative ability of allele sequences versus SNPs to detect selection, and in the environmental factors imposing selective pressures on these genes [77]. However, our analyses of SNP markers are consistent with other data sets indicating that in *Peromyscus*, mating behavior is associated with patterns of immunogenetic diversity. In particular, locus-specific studies have revealed greater MHC diversity [9] and gene expression analyses have demonstrated upregulation of MHC genes [11] in polygynandrous relative to monogamous species in this genus. Thus, while much remains to be learned regarding the mechanisms (e.g., mate choice) by which reproductive behavior contributes to observed differences in MHC variation, it seems increasingly apparent that mating system is an important driver of immunogenetic diversity in these animals.

### Future directions

Although our analyses suggest that mating systems are important contributors to patterns of immunogenetic variability, the effects of behavior on genetic diversity are embedded within a complex network of interactions involving elements of behavior, ecology, demography, and immunology. As a result, while patterns of mating behavior–in particular the number of reproductive partners per individual–appear to be important, there are clearly other factors that need to be considered to understand patterns of immunogenetic diversity in natural populations of animals. As a first step, it would be valuable to assess the generality of the relationships reported here. Thus, future studies should include *P*. *polionotus*–an independently evolved example of monogamy within the genus *Peromyscus* [78]; if comparisons of this species and sympatric, polygynandrous congeners reveal interspecific differences in immunogenetic diversity similar to those reported here, this replication would add strength to the assertion that mating systems are key determinants of immunogenetic diversity. At the same time, efforts to explore more directly interactions between mating behavior and specific immunogenes–including experimental manipulation of behavior, pathogen exposure, and immune response–should significantly improve understanding of why patterns of diversity vary among MHC loci as well as between these and other immunologically important loci. Finally, more detailed characterization of habitat parameters may identify important, behaviorally mediated correlates of pathogen exposure that were not detected in this study. Habitat conditions are expected to play a critical role in shaping both mating systems and pathogen exposure and thus efforts to integrate these themes more directly to examine impacts on immunogenetic diversity should generate new insights into relationships among environmental, phenotypic, and genomic variation.

## Supporting information

S1 Fig Estimates of per-site nucleotide diversity (π) for (A) SNPs associated with MHC loci and (B) all SNPs identified in each study species. Data for each population of conspecifics were analyzed separately; these data are summarized in Table 1. Species names are abbreviated and color coded as in the legend.
(TIF)

S1 Table NCBI RefSeq IDs and gene annotations for the 46 MHC Class I and II loci (including MHC regulatory factors and transactivator proteins) for which SNPs were identified. Shaded cells denote genes (N = 27) used in species-level estimates of genetic diversity; bolded cells denote genes (N = 13) associated with population-level estimates of diversity (See Table 1).
(DOCX)
