# Supplementary figures and images for: Mating system is correlated with immunogenetic diversity in sympatric species of Peromyscine mice
Source: PLoS One. 2020 Jul 23;15(7):e0236084. doi: 10.1371/journal.pone.0236084 (PMC7377423; doi:10.1371/journal.pone.0236084)

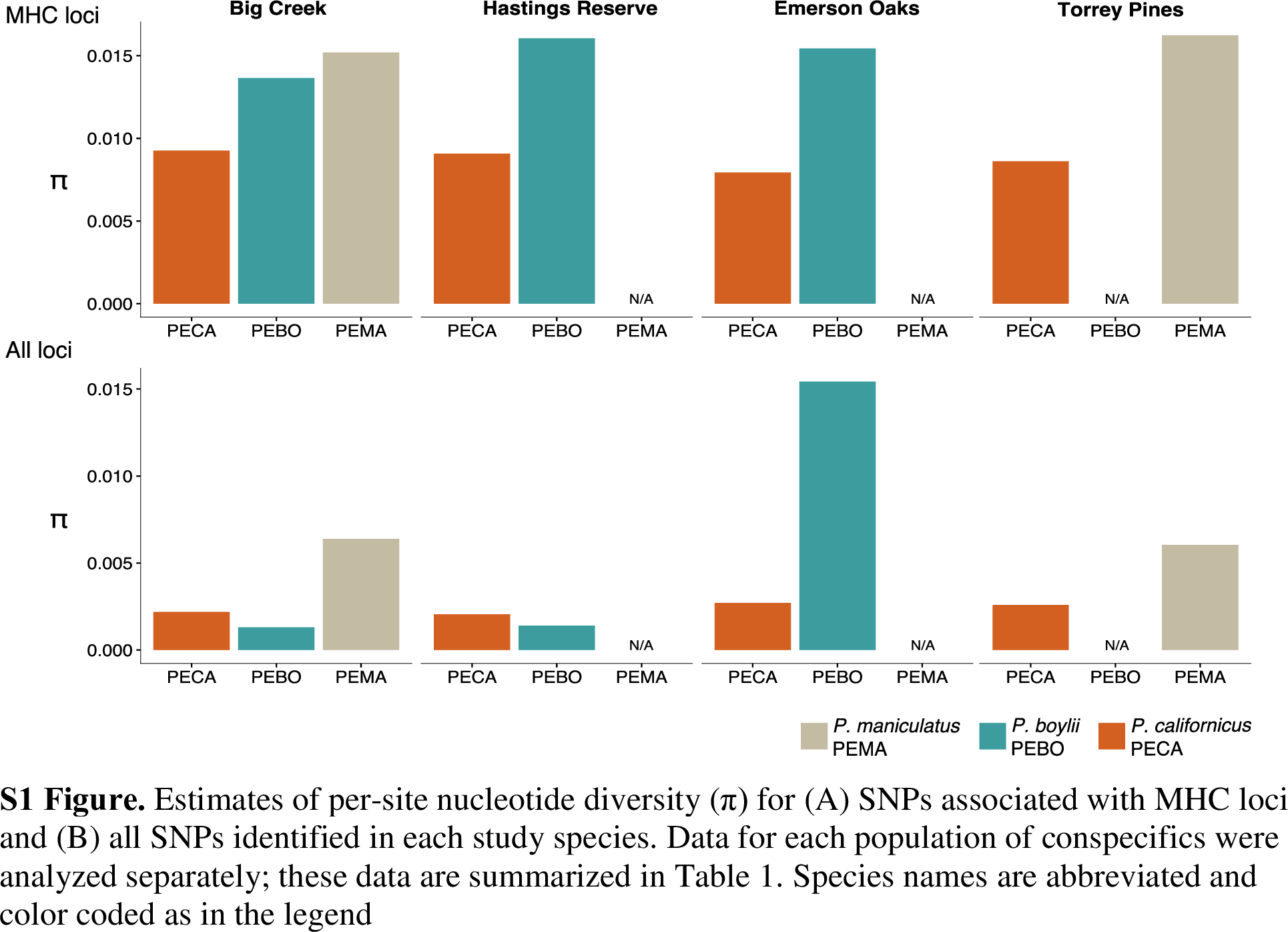

Supplement: S1 Fig — Estimates of per-site nucleotide diversity (π) for (A) SNPs associated with MHC loci and (B) all SNPs identified in each study species. Data for each population of conspecifics were analyzed separately; these data are summarized in Table 1. Species names are abbreviated and color coded as in the legend. (TIF) [file pone.0236084.s001.tif]
