# Supplementary material for: Mating system is correlated with immunogenetic diversity in sympatric species of Peromyscine mice
Source: PLoS One. 2020 Jul 23;15(7):e0236084. doi: 10.1371/journal.pone.0236084 (PMC7377423; doi:10.1371/journal.pone.0236084)
Supplement: S1 Table — Shaded cells denote genes (N = 27) used in species-level estimates of genetic diversity; bolded cells denote genes (N = 13) associated with population-level estimates of diversity (See Table 1). (DOCX) [file pone.0236084.s002.docx]

| **S1 Table.** NCBI RefSeq IDs and gene annotations for the 46 MHC Class I and II loci (including MHC regulatory factors and transactivator proteins) for which SNPs were identified. Shaded cells denote genes (N = 27) used in species-level estimates of genetic diversity; bolded cells denote genes (N = 13) associated with population-level estimates of diversity (See Table 1). | |
| --- | --- |
| **SeqName** | **Gene Annotation** |
| XM_006985129.2 | HLA class II histocompatibility antigen gamma chain isoform X1 |
| **XM_006993083.2** | **MHC class II regulatory factor RFX1** |
| XM_006995227.2 | H-2 class I histocompatibility Q10 alpha chain-like isoform X1 |
| XM_006996593.2 | major histocompatibility complex class I antigen |
| XM_006996816.2 | major histocompatibility complex class I antigen |
| XM_006997038.2 | MHC class II antigen [Rattus norvegicus] (*DQβ*) |
| XM_006997044.2 | MHC class II antigen [Mus musculus] |
| **XM_006997045.2** | **class II histocompatibility M alpha chain** |
| XM_006997281.2 | H-2 class I histocompatibility Q10 alpha chain isoform X2 |
| XM_006997312.2 | H-2 class I histocompatibility D-37 alpha chain-like |
| **XM_006997388.2** | **MHC class II antigen [Rattus norvegicus]** |
| XM_006997389.2 | MHC class II antigen [Rattus norvegicus] (*DQα*) |
| XM_006997392.2 | MHC class II antigen [Rattus norvegicus] |
| XM_006997711.2 | H-2 class I histocompatibility D-37 alpha chain-like isoform X1 |
| XM_006997991.2 | H-2 class I histocompatibility Q10 alpha chain-like isoform X1 |
| XM_006998077.2 | major histocompatibility complex class I antigen |
| XM_006998677.2 | H-2 class I histocompatibility Q10 alpha chain-like |
| XM_006998788.1 | H-2 class I histocompatibility Q9 alpha chain- partial |
| XM_006998845.2 | H-2 class I histocompatibility Q9 alpha chain- partial |
| XM_006999020.1 | H-2 class I histocompatibility Q10 alpha chain-like |
| XM_015988730.1 | H-2 class I histocompatibility Q10 alpha chain-like isoform X2 |
| XM_015990460.1 | H-2 class I histocompatibility Q10 alpha chain-like isoform X1 |
| XM_015990497.1 | MHC class II antigen [Rattus norvegicus] |
| XM_015990499.1 | MHC class II antigen |
| XM_015991363.1 | H-2 class I histocompatibility Q10 alpha chain-like isoform X1 |
| XM_015991686.1 | H-2 class I histocompatibility Q9 alpha chain- |
| XM_015996941.1 | minor histocompatibility HA-1 isoform X1 |
| **XM_016004093.1** | **major histocompatibility complex class I-related gene** |
| XM_006998063.2 | major histocompatibility complex class I antigen |
| XM_015991209.1 | patr class I histocompatibility A-126 alpha chain-like isoform X1 [Peromyscus maniculatus bairdii] |
| XM_006998564.2 | H-2 class I histocompatibility Q10 alpha chain-like |
| **XM_016002443.1** | **MHC class II transactivator isoform X1 (*Ciita*)** |
| XM_006996074.1 | HLA class I histocompatibility A-36 alpha chain-like |
| XM_006997391.2 | H-2 class II histocompatibility E-D beta chain-like |
| XM_006997393.2 | H2-Eb1 [Mus musculus] (*DRβ)* |
| XM_015991362.1 | H-2 class I histocompatibility Q10 alpha chain-like isoform X1 |
| XM_006997048.2 | HLA class II histocompatibility DO alpha chain |
| XM_015991681.1 | H-2 class I histocompatibility Q10 alpha chain-like |
| **XM_006985130.2** | **HLA class II histocompatibility antigen gamma chain isoform X2** |
| **XM_006996070.1** | **saoe class I histocompatibility A alpha chain-like** |
| **XM_015989455.1** | **H-2 class I histocompatibility Q10 alpha chain-like** |
| **XM_015989456.1** | **MHC class Ib antigen short [Rattus norvegicus]** |
| **XM_015989911.1** | **H-2 class I histocompatibility Q10 alpha chain-like isoform X2** |
| **XM_015990243.1** | **HLA class II histocompatibility DO beta chain** |
| **XM_015990498.1** | **MHC class II antigen [Rattus norvegicus]** |
| **XM_015991210.1** | **patr class I histocompatibility A-126 alpha chain-like iso** |
